# Heavy Metal Accumulation in Oysters from an Aquaculture Area in the Luoyangjiang River Estuary

**DOI:** 10.3390/toxics12090645

**Published:** 2024-08-31

**Authors:** Yizhou Ke, Changchun Ou, Xiaoyu Guo, Shuyi Liu, Chenlu Yao, Bo Shi, Huayong Que

**Affiliations:** 1 Key Laboratory of Healthy Mariculture for the East China Sea, Ministry of Agriculture and Rural Affairs, Jimei University, Xiamen 361021, China; organic_cycle@outlook.com (C.O.); lsy00410@163.com (S.L.); yaochenlu0131@163.com (C.Y.); shibo@jmu.edu.cn (B.S.); 2 State Key Laboratory of Mariculture Breeding, Fisheries College of Jimei University, Xiamen 361021, China; 3 College of Oceanology and Food Science, Quanzhou Normal University, Quanzhou 362000, China; guowen1999@163.com

**Keywords:** oyster, heavy metal, accumulation, estuary

## Abstract

Oysters are a group of economically important bivalves in China, with estuaries serving as one of their primary cultivation areas. However, heavy metal pollution in these estuarine environments poses a potential threat to aquaculture by leading to the accumulation of heavy metals in farmed oysters, which could impact their safety and marketability. This study was conducted in the aquaculture area of the Luoyangjiang River estuary, where eight sampling sites were selected. Water, sediment, and oysters categorized by shell length were collected from each site. The concentrations of heavy metals (Ag, As, Cd, Cr, Cu, Ni, Pb, and Zn) were determined in both the environmental samples and oyster tissues. Additionally, multiplex species-specific PCR was used to identify oyster species. The results showed significant variations in dissolved-phase and suspended particulate matter (SPM) metal concentrations across different sampling sites, while sediment metal concentrations were more consistent but similar to those in SPM. The large oysters were comprised of 50% *Magallana angulata* and 50% *Magallana gigas*, while small oysters were identified as *Magallana sikamea*. The Cd, Cu, Pb, and Zn levels in both size groups of oysters exceeded data from previous studies, indicating contamination in the estuary. The observed differences in heavy metal concentrations between large and small oysters primarily reflect species-specific variability in metal accumulation, which may also be influenced by factors such as growth and exposure duration. Furthermore, the lack of significant correlation between metal concentrations in environmental media and oysters suggests that oysters may be exposed to multiple sources of metal contamination.

## 1. Introduction

Over the past several decades, numerous reports have documented heavy metal pollution in China’s estuaries, encompassing the estuarine waters, sediments, and biota [1,2,3]. In recent years, due to the active efforts of the government, there has been a reduction in both the number and severity of these reports [4,5]. However, estuaries serve as critical interfaces between riverine and marine ecosystems, and their complex hydrodynamic and sedimentary processes make them particularly vulnerable to heavy metal pollutants, thereby posing a persistent ecological risk [6,7]. Along with economic development, human activities—particularly industrial discharges, urban runoff, and agricultural practices—still caused metal pollution, especially in estuarine areas. 

China is the leading producer of mollusks globally, accounting for most of the world’s shellfish output, with bivalves representing a significant share of this production [8,9]. Oysters are a group of traditional aquaculture species in China, also with the largest annual production in the world: approximately 6.7 million tons in 2023 [10]. Oysters are predominantly cultured in the rich, nutrient-dense waters in coastal seas or estuaries [11]. Consequently, the presence of heavy metals in these zones may pose risks to farmed oysters and threaten human health through their consumption [12,13]. Thus, understanding the impact of metal pollution on farmed oysters in these areas is crucial for sustainable environmental management and the protection of public health.

As filter-feeding organisms, bivalves can absorb heavy metals in their tissues through both dissolved and particulate phases under natural conditions [14,15], and they usually possess a higher capacity to accumulate these metals from aquatic environments compared to many other large aquatic organisms [16]. Additionally, it is worth noting that oysters exhibit a remarkable propensity for accumulating metals such as Cu and Zn [17,18], which consequently increases their ecological risk in environments contaminated with heavy metals. 

Due to their high-level metal bioaccumulation abilities and sessile lifestyle, oysters are also frequently employed in biomonitoring programs to assess the health of aquatic ecosystems, particularly in areas impacted by metal pollution [19,20,21]. Significant differences in heavy metal accumulation have been observed among different species of oysters, as confirmed by both laboratory and field studies [22,23]. For wild oysters, distinguishing specific species based solely on physical appearance is often challenging due to their morphological plasticity [24]. Historically, most field studies on metal pollution have not employed advanced techniques such as molecular technology that are capable of accurately identifying oyster species.

The primary objective of this study was to investigate the types of metals contaminating the aquaculture area and to understand the differences in metal accumulation across various oyster species, focusing on a specific set of heavy metals—silver (Ag), arsenic (As), cadmium (Cd), chromium (Cr), copper (Cu), nickel (Ni), lead (Pb), and zinc (Zn)—commonly found as pollutants in China’s coastal areas. Environmental samples (water and sediment) and oysters of two different sizes were collected from eight sites within an oyster farming area in the Luoyangjiang River estuary. Both dissolved and particulate metal concentrations in the water as well as in the sediment and oysters were determined. The correlation between heavy metal concentrations in the environmental samples and those in the oysters was analyzed. In addition, multiplex species-specific PCR identification was used to confirm the species of the collected oysters. Our study is valuable for developing effective management and conservation strategies to protect estuary ecosystems and ensure sustainable bivalve farming. 

## 2. Materials and Methods

### 2.1. Sampling Sites

**Figure 1:**
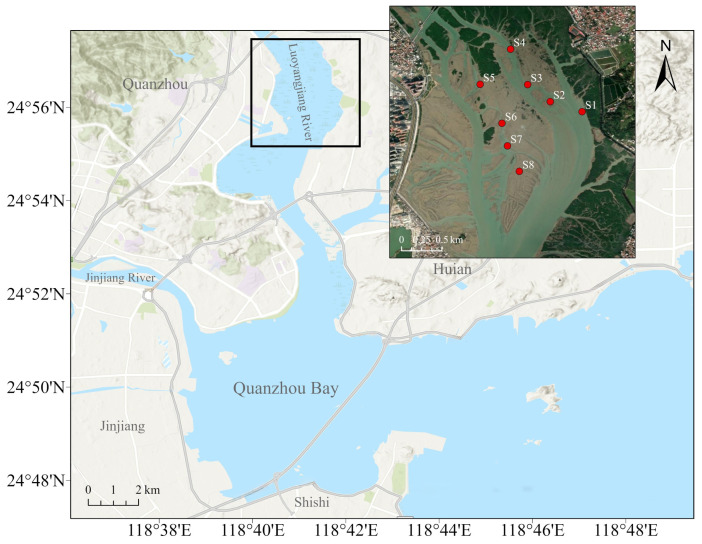
Sampling sites for oyster collection in the Luoyangjiang River estuary area. Eight sampling sites (S1 to S8) where water, sediment, and oyster samples were collected.

Considering the range of shellfish-farming areas along the coast of the Luoyangjiang River estuary, Quanzhou, Fujian, China, a representative farming area at the estuary was selected (Figure 1). Semi-artificial collection of oyster spat in this area involves placing artificial collectors in natural oyster spawning grounds to enhance the settlement and collection of oyster larvae/spat. In November 2022, eight sites within the Luoyangjiang estuary farming area were sampled during the low-tide period within one day. A total of eight sampling sites are shown in Figure 1, marked as S1 to S8. Salinity, dissolved oxygen, pH, and temperature were measured at each sample site (YSI-ProQuatro, Yellow Springs, OH, USA). Meanwhile, water, sediment, and oyster samples were collected at each site.

### 2.2. Collection and Processing of Water and Sediment Samples

Sampling bottles (500 mL polyethylene bottles) were acid-washed prior to sampling (soaked in 5% HNO_3_ for at least 24 h), rinsed three times with Milli-Q water, and then stored in sealed bags for use. Before collecting water samples, the bottles were rinsed three times with seawater from the sampling site. The polyethylene bottles were submerged about 10 cm into the seawater to collect 500 mL water samples, which were then transported back to the laboratory within 4 h. Each water sample was filtered through a pre-weighed 0.45 μm polysulfone filter to separate the dissolved and particulate parts. Filtered water was transferred to polystyrene tubes. The samples were stored at 4 °C before further analysis. The suspended particulate matter (SPM), along with the filter membrane, was dried at 80 °C for 48 h until a constant weight was achieved, and the dry weight was recorded. Filter membranes and the retained SPM (0.02–0.10 g) were digested in a closed polytetrafluoroethylene vessel using a mixture of 9 mL concentrated HNO_3_ and 3 mL concentrated HCl. To ensure quality assurance, procedural blanks (membrane without particles) were processed and analyzed alongside the samples. After digestion, the SPM solutions were centrifuged at 3000× *g* for 5 min, and the supernatant was collected for metal analysis. 

At each site, approximately 500 g of surface sediment (top 10 cm) was collected, placed in sealed bags, and transported back to the laboratory, where they were stored at −20 °C until the beginning of the procedure. The sediments were wet-sieved through a 63 μm nylon mesh, and the fraction smaller than 63 μm was dried in an oven at 80 °C until a constant weight was achieved. The dried sediment was ground into a powder, and approximately 0.5 g of sediment from each site was weighed three times and placed in 15 mL tubes. The samples were processed following the same procedure as the SPM samples for the remaining steps.

### 2.3. Collection and Processing of Oysters

About 30–40 individual oysters, including both small and large ones, were collected at each site after the environmental samples were gathered. The collected oysters were placed in sealed bags and transported to the laboratory within 4 h. The shell length of oysters was measured at each sampling site, with 10 large and 10 small oysters randomly selected per site. The oysters were cleaned and stored at −20 °C before dissection. For each sample site, 10 small and 10 large individuals were dissected by scalpel, and the soft tissues were collected and dried using a freeze dryer for 48 h to remove moisture. After drying, the dry weight was measured and recorded. The tissues were placed into corresponding 15 mL polystyrene tubes. Each tube was then added with 1 mL of 65% HNO_3_ and sequentially heated at 80 °C for 8 h. The samples were then diluted with Milli-Q water to appropriate concentrations in new polystyrene tubes. In addition, the remaining small and large oysters collected from all sites were separately combined, and from each mixture, adductor muscle tissues from 10 oysters were randomly selected for oyster species identification.

### 2.4. Identification of Oyster Species

To extract genomic DNA, approximately 15–20 mg of tissue was collected from the adductor muscle of oysters. The DNA extraction was performed using the TIANamp Marine Animal DNA Kit (Tiangen Biotech Co., Ltd., Beijing, China) according to the manufacturer’s protocol. Species identification was conducted using multiplex species-specific PCR (polymerase chain reaction). Amplification utilized the external COI universal primers LCO1490 (5′-GGTCAACAAATCATAAAGATATTGG-3′) and HCO2198 (5′-AAACTTCAGGGTGACCAAAAAATCA-3′), along with five Tridacna genus-specific primers (Table S1). The PCR reaction mixture consisted of a total volume of 25 µL, including 0.5 µL each of the external primers LCO1490 and HCO2198, 0.4 µL each of the five internal primers, 1.5 µL of DNA template, 12.5 µL of 2× PCR master mix (Vazyme Biotech Co., Ltd., Nanjing, China), and 8 µL of ddH_2_O to reach a final volume of 25 µL. The cycling parameters were as follows: initial denaturation at 95 °C for 5 min; 30 cycles of denaturation at 95 °C for 30 s, annealing at 48 °C for 1 min, and extension at 70 °C for 1 min; followed by a final extension at 72 °C for 10 min and preservation at 4 °C. Two microliters of the multiplex species-specific PCR reaction mixture was analyzed by 1.5% agarose gel electrophoresis, run at 120 V for 25 min. A 1000 bp marker (Axygen Biosciences, Union City, CA, USA) was used as a reference for determining the fragment sizes post-electrophoresis.

### 2.5. Chemical Analysis 

Metal concentrations in seawater, SPM, sediment, and oysters were determined using ICP-MS (Agilent 7800, Agilent Technologies, Santa Clara, CA, USA). This study measured a total of eight metals and metalloids: Ag, As, Cd, Cr, Cu, Ni, Pb, and Zn. Calibration of the ICP-MS was performed using internal standards (^72^Ge and ^118^In) to correct for instrumental drift and sensitivity fluctuations. Quality control samples were measured after every ten samples, with relative standard deviations maintained below 10%. The recovery rates for all metals of standard reference material SRM 1566b (oyster tissue), produced by the National Institute of Standards and Technology (NIST), were within a 10% deviation from the certified values. 

### 2.6. Calculation and Statistical Analysis

The data were analyzed using R software version 4.3.2. The partition coefficient (Kd) was determined by calculating the ratio of metal concentrations in particulate matter to those in the dissolved phase. A *t*-test was employed to assess differences in the results, with a significance level set at *p* < 0.05. Additionally, Pearson’s linear regression analysis was conducted to examine the relationship between heavy metal concentrations in environmental samples and those in oysters, with the same significance threshold of *p* < 0.05.

## 3. Results and Discussion

Our study focused on the estuarine intertidal zone within the oyster farming areas of the Luoyangjiang River estuary in China (Figure 1). In this area, oysters naturally recruit without the need for artificial intervention, as this method of natural spat collection is still used in China. The aquaculture area, surrounded by mangrove wetlands and influenced by human activities [25], provides a unique environment for investigating the impact of anthropogenic pollution on heavy metal accumulation in economically important bivalves. 

### 3.1. Hydrological Conditions 

The hydrological parameters measured at the eight sample sites in the Luoyangjiang River estuary, including temperature, pH, salinity, and dissolved oxygen (DO), are summarized in Table 1. The temperature across all sites ranged from 20.3 °C to 20.5 °C, indicating relatively stable thermal conditions within the sample area. The pH values varied slightly, with measurements between 7.66 and 7.79, suggesting a generally neutral to slightly alkaline environment. Salinity levels showed some variability, ranging from 23.1 psu to 24.6 psu, reflecting the influence of both freshwater input and tidal mixing. Dissolved oxygen concentrations were measured between 6.97 mg L⁻^1^ and 7.28 mg L⁻^1^, indicating well-oxygenated waters throughout the sampling sites. Varying hydrological conditions can significantly influence the accumulation of heavy metals in oysters. For example, higher temperatures, lower salinity, acidic pH, and hypoxic conditions may increase the bioavailability and uptake of metals, leading to higher accumulation levels in oyster tissues [26,27,28,29]. Since the hydrological conditions across the sampling sites showed minimal overall differences, their impact on the variation in metal accumulation among oysters at different sites is likely small.

### 3.2. Heavy Metals Concentrations in Water and Sediment

**Figure 2:**
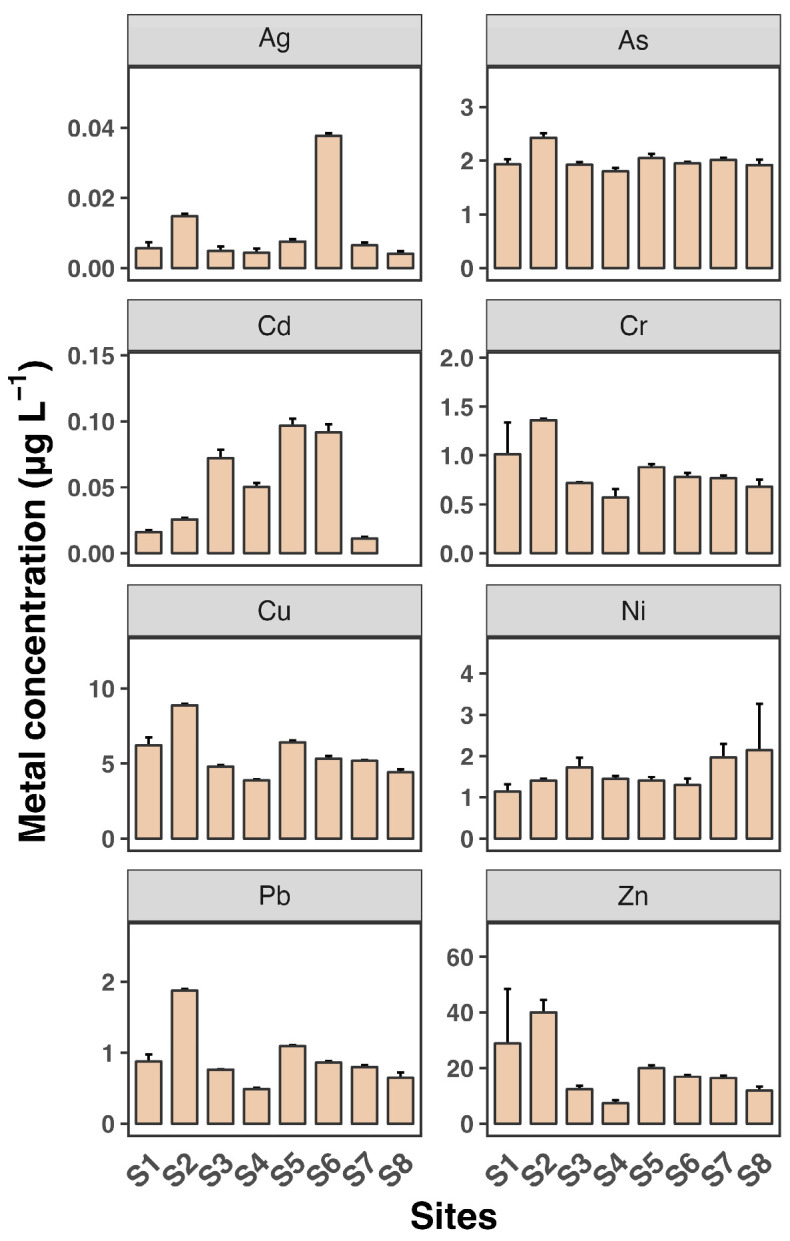
Dissolved concentrations of eight heavy metals (Ag, As, Cd, Cr, Cu, Ni, Pb, and Zn) in water samples from eight sampling sites (S1 to S8). Each sampling site includes three replicates. Data shown as mean ± SD (*n* = 3).

**Figure 3:**
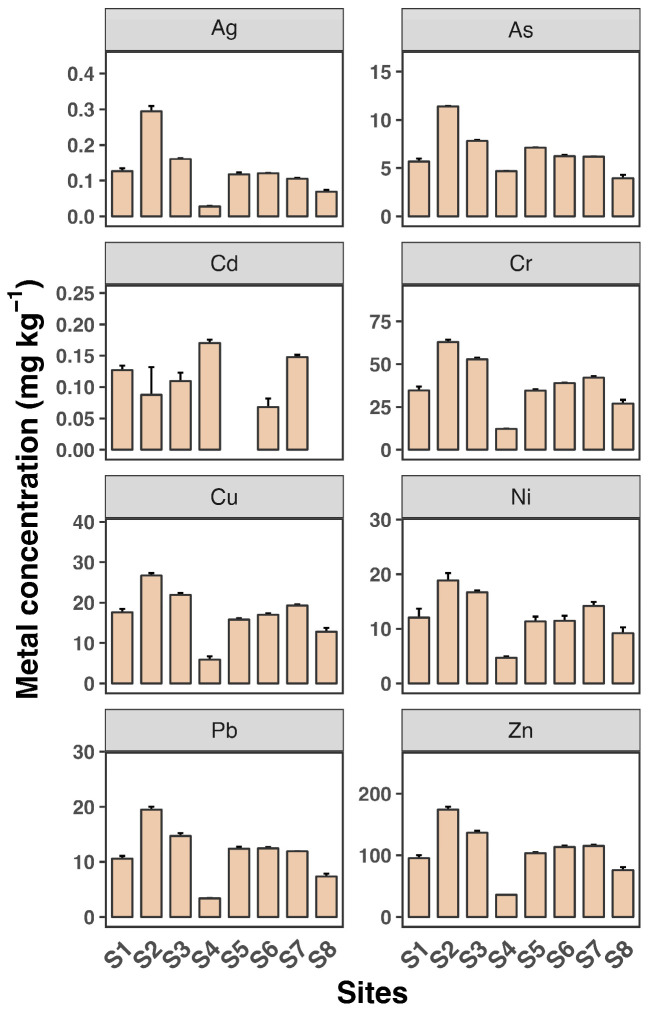
Suspended particle concentrations of eight heavy metals (Ag, As, Cd, Cr, Cu, Ni, Pb, and Zn) in water samples from eight sampling sites (S1 to S8). Each sampling site includes three replicates. Data shown as mean ± SD (*n* = 3).

Figure 2 and Figure 3 depict the dissolved and SPM metal concentrations across all sampling sites. All metals generally exhibited significantly higher levels in SPM compared to dissolved phases, indicating that particulate-bound transport is a major pathway for these metals in the estuary. Ag and Cd concentrations were generally low across all sites, with mean values not exceeding 0.04 µg L^−1^ and 0.1 µg L^−1^, respectively, consistent with findings from previous studies [30,31]. Zn levels were particularly high, reaching up to 40 µg L^−1^, with a maximum observed at site S2. The distribution of all metals in the dissolved phase across the eight sampling sites was similar, except for Cd. Consistent with the dissolved phase, Zn had the highest concentration among the metals, reaching up to 174 mg kg^−1^ in SPM. The high levels of Zn, particularly in SPM, are consistent with other research findings [7]. Estuaries often receive significant inputs of Zn from industrial and urban sources, leading to elevated concentrations in sediments and particulates.

Most metals showed slightly elevated levels at sites S2 and S3 in both the dissolved and SPM phases. Additionally, Cu and Cr as well as Pb and Zn exhibited very similar patterns across the sampling sites, suggesting a common source for Cu and Cr and for Pb and Zn, likely from the surrounding coastline. In contrast, As, Cd, and Ni showed minimal variation between sites, indicating that the source of these metals may be more upstream in the river. These findings reveal a heterogeneous distribution of dissolved and SPM heavy metals across the eight sampling sites in the Luoyangjiang River estuary. The heterogeneous distribution of heavy metals in estuarine environments is a common observation. Factors such as hydrodynamic conditions, sediment composition, and proximity to pollution sources contribute to this variability. For example, the Pearl River estuary shows similar patterns of metal distribution influenced by hydrological connectivity and estuarine mixing [6]. 

**Figure 4:**
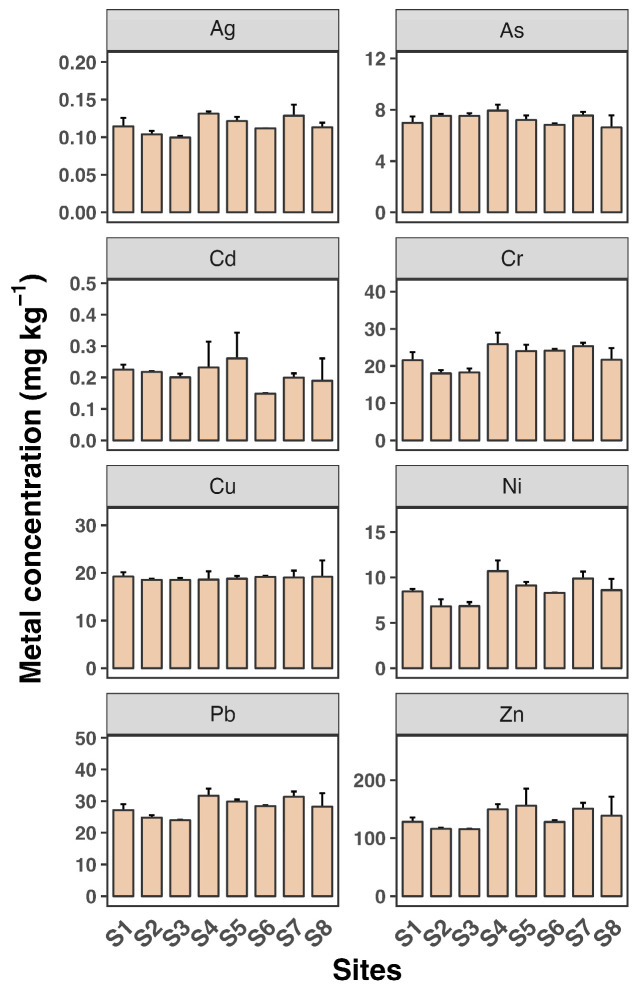
Sediment concentrations of eight heavy metals (Ag, As, Cd, Cr, Cu, Ni, Pb, and Zn) in water samples from eight sampling sites (S1 to S8). Each sampling site includes three replicates. Data shown as mean ± SD (*n* = 3).

Sediment concentrations were relatively stable across the sampling sites (Figure 4). The sediment analysis revealed significantly higher heavy metal accumulation compared to water and SPM, with maximum concentrations observed for Zn (over 22.6 mg kg^−1^), followed by Zn at over 1500 µg g^−1^ at site S8. The concentrations of Cd, Cu, Pb, and Zn were higher in sediments than in SPM, suggesting that these metals are likely pollution indicators. Although surface sediment concentrations were more stable compared to water, seasonal changes can lead to the resuspension of sediments and redistribution of metals. The hydrodynamics of estuarine systems, including tidal actions and riverine inputs, also contribute to the variability in metal concentrations [32,33].

### 3.3. Heavy Metals Accumulation in Oysters

Due to the significant size differences, with large oysters averaging 18.3 ± 4.1 cm and small oysters averaging 6.6 ± 2.5 cm, we categorized the oysters into two groups based on shell length. To understand the species of these two size groups of oysters, we identified the species collected from the sample sites using multiplex species-specific PCR technology, which has been successfully used to identify oyster and other bivalve species [34,35]. Figures S1 and S2 show that 100% of the small oysters were identified as *Magallana sikamea*, while the large oysters were a mix of 50% *Magallana angulata* and 50% *Magallana gigas*. In field experiments requiring oyster collection, it is common to encounter oysters of different species at the same sampling site [36]. However, some oyster species cannot be easily distinguished based on morphology alone, necessitating the use of molecular techniques for accurate identification. Therefore, during field oyster sample collection, it is essential to conduct species identification to enhance the accuracy of the conclusions.

**Figure 5:**
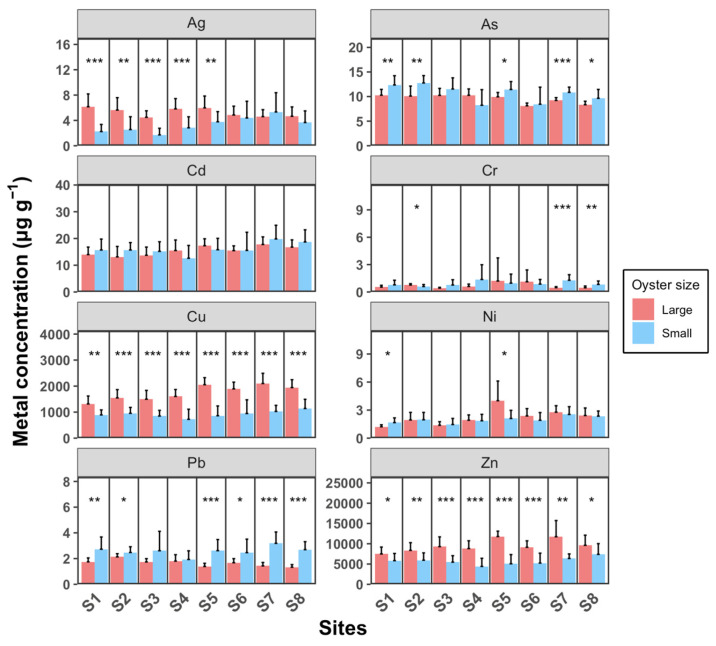
Oyster concentrations of eight heavy metals (Ag, As, Cd, Cr, Cu, Ni, Pb, and Zn) in water samples from eight sampling sites (S1 to S8). Each sampling site includes three replicates. Data are presented as mean ± SD (*n* = 10). Statistical significance is indicated as follows: *** *p* < 0.001; ** *p* < 0.01; * *p* < 0.05.

Figure 5 illustrates that larger oysters generally accumulate higher concentrations of Ag, Cu, and Zn compared to smaller individuals, with the exception of Pb, where smaller oysters show higher levels. For As, Cd, Cr, and Ni, there are generally no significant differences between the two size groups. The concentration ranges for Cd, Cu, Pb, and Zn across sampling sites are as follows: Cd ranges from 13.1 to 17.8 µg g^−1^ in large oysters and 12.6 to 19.8 µg g^−1^ in small oysters; Cu ranges from 1314 to 2100 µg g^−1^ in large oysters and 723 to 1139 µg g^−1^ in small oysters; Pb ranges from 1.33 to 2.13 µg g^−1^ in large oysters and 1.93 to 3.19 µg g^−1^ in small oysters; and Zn ranges from 7497 to 11,784 µg g^−1^ in large oysters and 4409 to 7387 µg g^−1^ in small oysters. Weng and Wang (2014) [23] compared metal concentrations in *M. sikamea* between two sampling sites with different pollution levels. For Cd, Cu, Pb, and Zn, the concentrations at the clean site were 2.4–5.8, 200–510, 0.75–1.5, and 900–510 µg g^−1^, respectively. In contrast, at the polluted site, the concentrations were significantly higher, in ranges of 6.1–16, 1900–5900, 1.9–3.4, and 3700–11,000 µg g^−1^, respectively. These concentrations at the polluted site are comparable to the levels observed in our study. Wang et al. (2011) [37] reported that in a polluted estuary, Cd, Cu, Pb, and Zn in *M. angulata* were detected in ranges of 7.8–49, 692–8846, 1.6–7.5, and 4692–24,200 µg g^−1^, respectively. For Ag, As, Cr, and Ni, the concentrations observed in our study are relatively similar to those reported in previous studies conducted in unpolluted areas [38]. Overall, our results suggest that our study area is specifically polluted with Cd, Cu, Pb, and Zn.

When heavy metals accumulate in bivalves beyond certain thresholds, they can exert toxic effects that impact the growth and reproduction of these organisms [39]. For example, Ag concentrations reaching 106 μg g^−1^ and Cu concentrations at 287 μg g^−1^ in *Macoma balthica* can significantly impair growth and gonadal development, resulting in reproductive failure during periods of high metal accumulation [40]. For oysters, no studies have demonstrated that heavy metal accumulation affects their growth; however, some studies have shown that excessive accumulation of heavy metals such as Cd, Cu, and Zn can adversely impact gonadal development [41,42].

Ag concentrations in large oysters are significantly higher than in small oysters at sample sites S1–S5 (*t*-test, *p* < 0.05). Specifically, in sites S1–S4, large oysters exhibit significantly higher Ag levels compared to small oysters, whereas this difference is not observed at sites S6–S8. The lower Ag concentrations in small oysters from sites S1–S4 compared to those from sites S5–S8 suggest that the primary source of Ag may be located on the west shore of the estuary. Cu and Zn concentrations in large oysters are significantly higher than in small oysters across all sampling sites (*t*-test, *p* < 0.05). Interestingly, Pb is the only metal where small oysters consistently show significantly higher concentrations than large oysters at all sampling sites (*t*-test, *p* < 0.05). 

There are several reasons that explain the differences between the different-sized oysters in our study. The primary reason is that some oyster species have a unique mechanism for accumulating Cu and Zn. Differences in biokinetic parameters between oyster species may explain to these variations. The elimination rate constant $k_{e}$s for Cu and Zn in *M. angulata* and *M. gigas* is nearly 0 d^−1^ [20], indicating that these species can barely efflux Cu and Zn. In contrast, studies have reported that the $k_{e}$s for Cu and Zn in *M. sikamea* is around 0.08 d^−1^, suggesting that *M. sikamea* is capable of effluxing a portion of these metals. Notably, the $k_{e}$s for Cd in both *M. angulata* and *M. sikamea* is also close to 0 d^−1^ [20,43]. These findings suggest that the primary factors controlling interspecies differences in heavy metal accumulation in oysters are physiological in nature.

**Figure 6:**
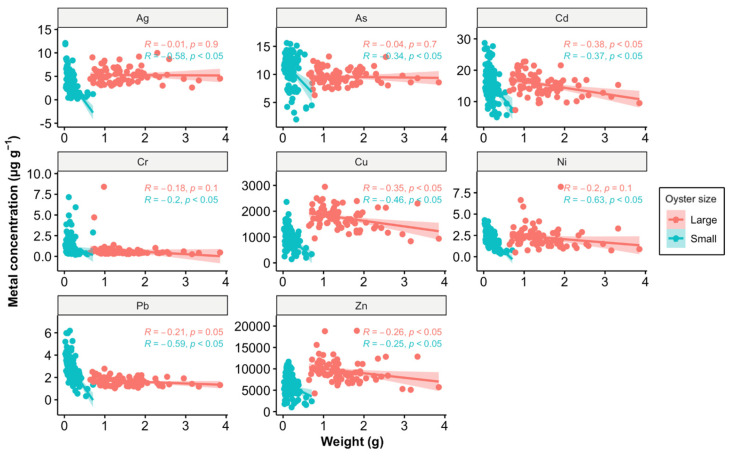
Relationship between metal concentration (µg g^−1^) and weight (g) in oysters, analyzed using Pearson correlation. The data points are divided into two size categories: large (red) and small (blue). The Pearson correlation coefficients (*R*) and *p*-values (*p*) are provided for each size category, indicating the strength and significance of the relationship. A *p*-value of less than 0.05 suggests that the correlations are statistically significant. The shaded areas around the regression lines represent the 95% confidence intervals.

Another possible explanation could be the growth dilution effect (Figure 6). Among the eight metals analyzed in this study, Cd, Cu, and Zn—identified as pollutants in the estuary—showed significant correlations between weight and metal concentration in tissues in large oysters, while small oysters exhibited significant correlations with all eight metals. As oysters grow, their increasing body mass can dilute the concentration of accumulated metals in their tissues. Typically, smaller oysters tend to have higher concentrations of certain metals due to their higher metabolic and growth rates, which can lead to greater uptake of metals from the environment [44]. However, our findings reveal that only Pb concentrations were higher in small oysters compared to large ones, suggesting that the dilution effect alone does not fully account for the observed data.

The final reason is that in our study, we did not know the exposure time of the oysters collected from the field. Generally, longer exposure times can lead to higher accumulation of heavy metals in bivalves. It is worth noting that previous studies have reported that bivalves can regulate metal concentrations within their bodies. For example, the razor clam *Sinonovacula constricta* has a mechanism for regulating the accumulation of metals such as Cu during long-term exposure [45]. This regulatory capability in bivalves is limited when metal concentrations in water exceed a certain threshold [46,47]. However, there are limited reports specifically addressing this mechanism in oysters. 

**Figure 7:**
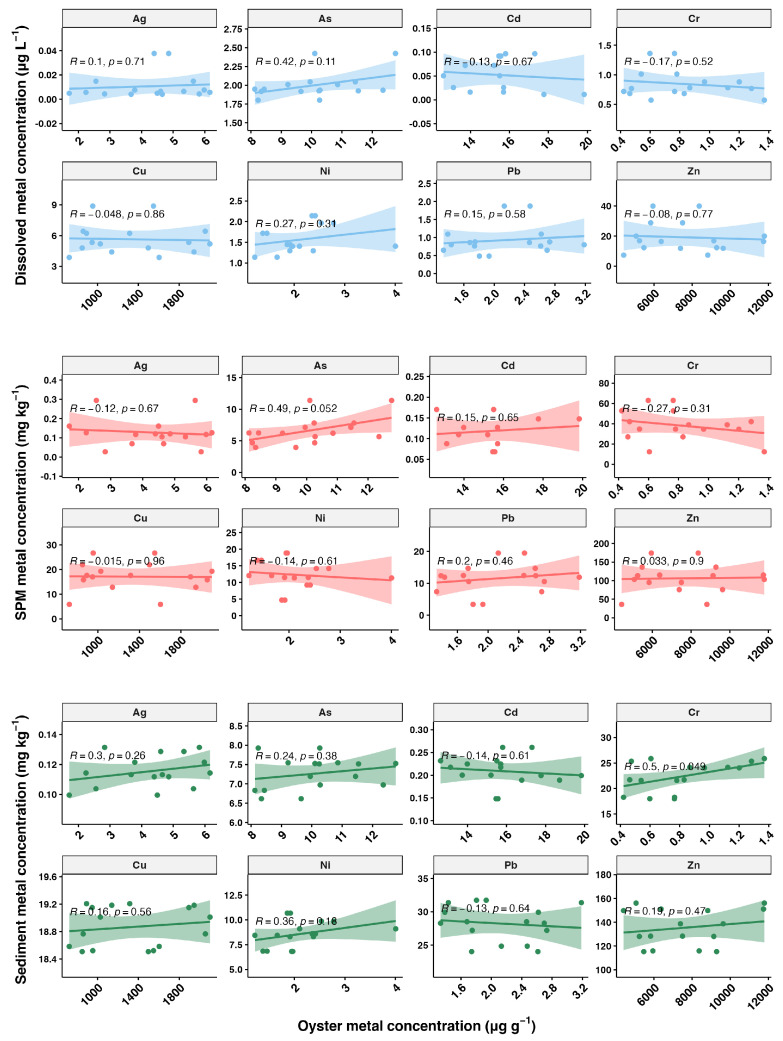
Relationship between oyster metal concentrations (µg g⁻^1^) and metal concentrations in the environment. The plots are divided into three rows representing different environmental matrices: dissolved phase, suspended particulate matter (SPM), and sediment. The scatter plots show the correlation between the metal concentrations in oysters and the corresponding environmental matrix, with Pearson correlation coefficients (*R*) and *p*-values (*p*) indicated in each plot. A *p*-value of less than 0.05 suggests that the correlations are statistically significant. The shaded areas around the regression lines represent the 95% confidence intervals.

The relationship between metal concentrations in oysters and those in the environment is depicted in Figure 7, showing a weak correlation. Although we conducted sampling during low tide, pollutant discharge may be intermittent and without a consistent release pattern. In field studies, it is generally difficult to determine whether the heavy metals accumulated in bivalves primarily originate from the dissolved phase or the particulate phase. Laboratory studies have indicated that both pathways can significantly contribute to heavy metal accumulation in bivalves. Therefore, when interpreting the accumulation patterns of heavy metals in bivalves, it is crucial to consider the contributions of both dissolved and particulate routes [15]. Overall, our results indicate that biomonitoring offers a more reliable assessment of heavy metal pollution compared to direct measurements of metal concentrations in environmental media.

## 4. Conclusions

Because the concentration fluctuations of heavy metals in environmental media can be relatively more variable compared to those in oysters, metal concentrations in oyster tissues provide a more accurate reflection of heavy metal pollution. In our study area of the Luoyangjiang River estuary, the primary pollutants identified were Cd, Cu, Pb, and Zn. In addition, differences in oyster species significantly impact the accumulation of heavy metals, a variation that may result from a combination of multiple factors, with the most important being the biological mechanisms of metal accumulation that vary between different species. Therefore, accurate identification of oyster species is essential, as the oyster serves as a reliable biomonitoring indicator in aquaculture farm areas. Our findings are valuable for mitigating risks associated with heavy metal contamination in bivalve aquaculture, ensuring the safety of seafood products and protecting the health of these ecologically and economically important farming areas. 

## Figures and Tables

**Table 1:** Hydrological parameters at eight sample sites in the Luoyangjiang River estuary, including temperature, pH, salinity, and dissolved oxygen (DO).

Sample Site	Temperature (°C)	pH	Salinity (‰)	DO (mg L^−1^)
S1	20.4	7.69	23.4	7.05
S2	20.4	7.77	23.3	7.22
S3	20.3	7.71	23.1	7.09
S4	20.4	7.78	24.6	7.26
S5	20.4	7.79	24.5	7.28
S6	20.5	7.66	24.2	6.97
S7	20.4	7.73	23.6	7.13
S8	20.5	7.78	23.1	7.26

## Data Availability

Data are contained within the article.

## Supplementary Materials

The following supporting information can be downloaded at https://www.mdpi.com/article/10.3390/toxics12090645/s1. Table S1: PCR primers of genus and species-specific and expected product size; Figure S1: Multiplex species-specific PCR identification results of small oysters; Figure S2: Multiplex species-specific PCR identification results of large oysters.

## References

[1] Bi S., Yang Y., Xu C., Zhang Y., Zhang X., Zhang X. (2017). Distribution of Heavy Metals and Environmental Assessment of Surface Sediment of Typical Estuaries in Eastern China. Mar. Pollut. Bull..

[2] Zhang M., Sun X., Xu J. (2020). Heavy Metal Pollution in the East China Sea: A Review. Mar. Pollut. Bull..

[3] Wang S.-L., Xu X.-R., Sun Y.-X., Liu J.-L., Li H.-B. (2013). Heavy Metal Pollution in Coastal Areas of South China: A Review. Mar. Pollut. Bull..

[4] Xue S., Jian H., Yang F., Liu Q., Yao Q. (2022). Impact of Water-Sediment Regulation on the Concentration and Transport of Dissolved Heavy Metals in the Middle and Lower Reaches of the Yellow River. Sci. Total Environ..

[5] Zhao Y., Wu R., Cui J., Gan S., Pan J., Guo P. (2020). Improvement of Water Quality in the Pearl River Estuary, China: A Long-Term (2008–2017) Case Study of Temporal-Spatial Variation, Source Identification and Ecological Risk of Heavy Metals in Surface Water of Guangzhou. Environ. Sci. Pollut. Res..

[6] Niu L., Cai H., Jia L., Luo X., Tao W., Dong Y., Yang Q. (2021). Metal Pollution in the Pearl River Estuary and Implications for Estuary Management: The Influence of Hydrological Connectivity Associated with Estuarine Mixing. Ecotoxicol. Environ. Saf..

[7] Jia Z., Li S., Liu Q., Jiang F., Hu J. (2021). Distribution and Partitioning of Heavy Metals in Water and Sediments of a Typical Estuary (Modaomen, South China): The Effect of Water Density Stratification Associated with Salinity. Environ. Pollut..

[8] Hu F., Zhong H., Wu C., Wang S., Guo Z., Tao M., Zhang C., Gong D., Gao X., Tang C. (2021). Development of Fisheries in China. Reprod. Breed..

[9] Cao L., Naylor R., Henriksson P., Leadbitter D., Metian M., Troell M., Zhang W. (2015). China’s Aquaculture and the World’s Wild Fisheries. Science.

[10] Fisheries and Fisheries Administration Bureau of the Ministry of Agriculture (2023). China Fishery Statistical Yearbook.

[11] Forrest B.M., Keeley N.B., Hopkins G.A., Webb S.C., Clement D.M. (2009). Bivalve Aquaculture in Estuaries: Review and Synthesis of Oyster Cultivation Effects. Aquaculture.

[12] Gao S., Wang W.-X. (2014). Oral Bioaccessibility of Toxic Metals in Contaminated Oysters and Relationships with Metal Internal Sequestration. Ecotoxicol. Environ. Saf..

[13] Liu J., Zhang J., Lu S., Zhang D., Tong Z., Yan Y., Hu B. (2020). Interannual Variation, Ecological Risk and Human Health Risk of Heavy Metals in Oyster-Cultured Sediments in the Maowei Estuary, China, from 2011 to 2018. Mar. Pollut. Bull..

[14] Wu X., Jia Y., Zhu H., Wang H. (2010). Bioaccumulation of Cadmium Bound to Humic Acid by the Bivalve *Meretirx Linnaeus* from Solute and Particulate Pathways. J. Environ. Sci..

[15] Wang W.-X. (2001). Comparison of Metal Uptake Rate and Absorption Efficiency in Marine Bivalves. Environ. Toxicol. Chem..

[16] Fukunaga A., Anderson M.J. (2011). Bioaccumulation of Copper, Lead and Zinc by the Bivalves *Macomona liliana* and *Austrovenus stutchburyi*. J. Exp. Mar. Biol. Ecol..

[17] Liu F., Wang W.-X. (2014). Differential Influences of Cu and Zn Chronic Exposure on Cd and Hg Bioaccumulation in an Estuarine Oyster. Aquat. Toxicol..

[18] Tan Q.-G., Wang Y., Wang W.-X. (2015). Speciation of Cu and Zn in Two Colored Oyster Species Determined by X-Ray Absorption Spectroscopy. Environ. Sci. Technol..

[19] Zhou Q., Zhang J., Fu J., Shi J., Jiang G. (2008). Biomonitoring: An Appealing Tool for Assessment of Metal Pollution in the Aquatic Ecosystem. Anal. Chim. Acta.

[20] Cao X., Zhong G., Pan K., Qian J., Xie M., Chen R., Liao Y., Tan Q.-G. (2023). Interspecies Calibration for Biomonitoring Metal Contamination in Coastal Waters Using Oysters and Mussels. Sci. Total Environ..

[21] Liang L.N., HE B., Jiang G.B., Chen D.Y., Yao Z.W. (2004). Evaluation of Mollusks as Biomonitors to Investigate Heavy Metal Contaminations along the Chinese Bohai Sea. Sci. Total Environ..

[22] Liu X., Wang W.-X. (2016). Time Changes in Biomarker Responses in Two Species of Oyster Transplanted into a Metal Contaminated Estuary. Sci. Total Environ..

[23] Weng N., Wang W.-X. (2014). Variations of Trace Metals in Two Estuarine Environments with Contrasting Pollution Histories. Sci. Total Environ..

[24] Lam K., Morton B. (2006). Morphological and Mitochondrial-DNA Analysis of the Indo-West Pacific Rock Oysters (Ostreidae: *Saccostrea* Species). J. Molluscan Stud..

[25] Yang Y., Ye X., Wang A. (2023). Dynamic Changes in Landscape Pattern of Mangrove Wetland in Estuary Area Driven by Rapid Urbanization and Ecological Restoration: A Case Study of Luoyangjiang River Estuary in Fujian Province, China. Water.

[26] Lan W.-R., Huang X.-G., Lin L., Li S.-X., Liu F.-J. (2020). Thermal Discharge Influences the Bioaccumulation and Bioavailability of Metals in Oysters: Implications of Ocean Warming. Environ. Pollut..

[27] Cao R., Liu Y., Wang Q., Dong Z., Yang D., Liu H., Ran W., Qu Y., Zhao J. (2018). Seawater Acidification Aggravated Cadmium Toxicity in the Oyster Crassostrea Gigas: Metal Bioaccumulation, Subcellular Distribution and Multiple Physiological Responses. Sci. Total Environ..

[28] Sun M., Liu G., Lin H., Zhang T., Guo W. (2018). Effect of Salinity on the Bioaccumulation and Depuration of Cadmium in the Pacific Cupped Oyster, *Crassostrea gigas*. Environ. Toxicol. Pharmacol..

[29] Ivanina A.V., Froelich B., Williams T., Sokolov E.P., Oliver J.D., Sokolova I.M. (2011). Interactive Effects of Cadmium and Hypoxia on Metabolic Responses and Bacterial Loads of Eastern Oysters *Crassostrea virginica* Gmelin. Chemosphere.

[30] Sañudo-Wilhelmy S.A., Gill G.A. (1999). Impact of the Clean Water Act on the Levels of Toxic Metals in Urban Estuaries: The Hudson River Estuary Revisited. Environ. Sci. Technol..

[31] van Geen A., Luoma S.N. (1999). A Record of Estuarine Water Contamination from the Cd Content of Foraminiferal Tests in San Francisco Bay, California. Mar. Chem..

[32] Zhang Z., Jin J., Zhang J., Zhao D., Li H., Yang C., Huang Y. (2022). Contamination of Heavy Metals in Sediments from an Estuarine Bay, South China: Comparison with Previous Data and Ecological Risk Assessment. Processes.

[33] Sundaramanickam A., Shanmugam N., Cholan S., Kumaresan S., Madeswaran P., Balasubramanian T. (2016). Spatial Variability of Heavy Metals in Estuarine, Mangrove and Coastal Ecosystems along Parangipettai, Southeast Coast of India. Environ. Pollut..

[34] Ma H., Gao H., Zhang Y., Qin Y., Xiang Z., Li J., Zhang Y., Yu Z. (2021). Multiplex Species-Specific PCR Identification of Native Giant Clams in the South China Sea: A Useful Tool for Application in Giant Clam Stock Management and Forensic Identification. Aquaculture.

[35] Melo M.A.D., da Silva A.R.B., Beasley C.R., Tagliaro C.H. (2013). Multiplex Species-Specific PCR Identification of Native and Non-Native Oysters (Crassostrea) in Brazil: A Useful Tool for Application in Oyster Culture and Stock Management. Aquac. Int..

[36] Liu S., Xue Q., Xu H., Lin Z. (2021). Identification of Main Oyster Species and Comparison of Their Genetic Diversity in Zhejiang Coast, South of Yangtze River Estuary. Front. Mar. Sci..

[37] Wang L., Wang W. (2014). Depuration of Metals by the Green-colored Oyster *Crassostrea sikamea*. Environ. Toxicol. Chem..

[38] Lu G., Zhu A., Fang H., Dong Y., Wang W.-X. (2019). Establishing Baseline Trace Metals in Marine Bivalves in China and Worldwide: Meta-Analysis and Modeling Approach. Sci. Total Environ..

[39] Rainbow P.S. (2002). Trace Metal Concentrations in Aquatic Invertebrates: Why and so What?. Environ. Pollut..

[40] Hornberger M.I., Luoma S.N., Cain D.J., Parchaso F., Brown C.L., Bouse R.M., Wellise C., Thompson J.K. (2000). Linkage of Bioaccumulation and Biological Effects to Changes in Pollutant Loads in South San Francisco Bay. Environ. Sci. Technol..

[41] Weng N., Wang W.-X. (2015). Reproductive Responses and Detoxification of Estuarine Oyster *Crassostrea hongkongensis* under Metal Stress: A Seasonal Study. Environ. Sci. Technol..

[42] Weng N., Wang W.-X. (2019). Seasonal Fluctuations of Metal Bioaccumulation and Reproductive Health of Local Oyster Populations in a Large Contaminated Estuary. Environ. Pollut..

[43] Zhong G., Lin Z., Liu F., Xie M., Chen R., Tan Q.-G. (2024). Toxicokinetics and Mussel Watch: Addressing Interspecies Differences for Coastal Cadmium Contamination Assessment. Environ. Sci. Technol..

[44] Yang S., Li Y., Chen F., Chen S., Luo X., Duan W., Liao Y., Jiang H., Pan K. (2024). Understanding the Variable Metal Concentrations in Estuarine Oysters Crassostrea Hongkongensis: A Biokinetic Analysis. Mar. Environ. Res..

[45] Ke Y., Wang W.-X. (2023). Dynamics of Copper Regulation in a Marine Clam *Sinonovacula constricta* at the Organ Level: Insight from a Physiologically Based Pharmacokinetic Model. Environ. Pollut..

[46] Shi D., Wang W.-X. (2004). Understanding the Differences in Cd and Zn Bioaccumulation and Subcellular Storage among Different Populations of Marine Clams. Environ. Sci. Technol..

[47] Chan H. (1988). Accumulation and Tolerance to Cadmium, Copper, Lead and Zinc by the Green Mussel *Perna viridis*. Mar. Ecol. Prog. Ser..

