# Supplementary material for: Heavy Metal Accumulation in Oysters from an Aquaculture Area in the Luoyangjiang River Estuary
Source: Toxics. 2024 Aug 31;12(9):645. doi: 10.3390/toxics12090645 (PMC11436002; doi:10.3390/toxics12090645)
Supplement: Supplementary file 1 [file toxics-12-00645-s001.zip › toxics-3178394-supplementary.pdf]

Table S1. PCR primers of genus and species-specific and expected product size.

Primer	Specificity	Primer Sequence	Size (bp)
LCO1490	All		
COCar183r	*M. ariakensis*	AAAAAAGATTATAACTAATGCATGTCG(T)G	183
COCan222r	*M. angulata*	AGTTACCAAACCCCCCAATTATCAG(C)G	222
COCgi269r	*M. gigas*	TCGAGGAAATTGCATGTCTGCTACA(T)A	269
COChk387r	*M. hongkongensis*	GGAGTAAGTGGATAAGGGTGGATAG	387
COCsi546r	*M. sikamea*	AAGTAACCTTAATAGATCAGGGAAC(A)C	546
HCO2198	All		

*A mismatch nucleotide (bold) is introduced to promote specific amplification, and the correct base is presented in parentheses.

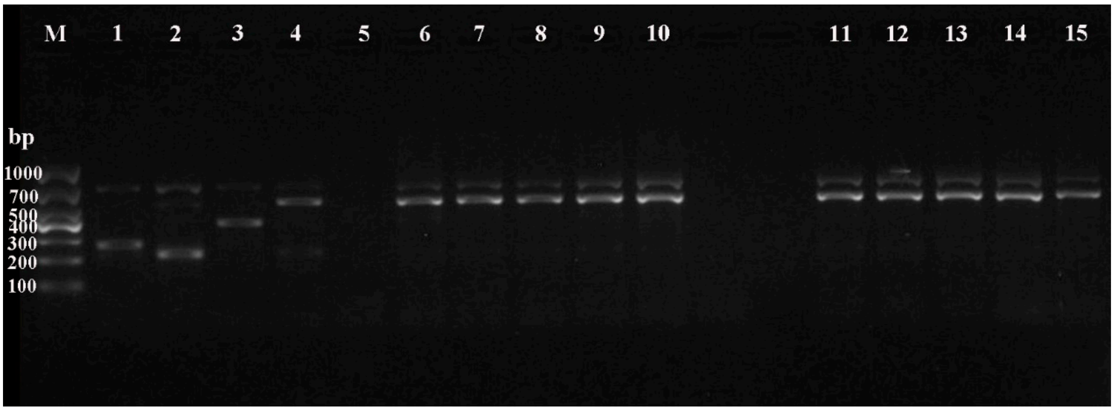

Figure S1. Multiplex species-specific PCR identification results of small oysters. Note: M. 1000 bp DNA Marker; 1. *Magallana gigas* (Standard Sample); 2. *Magallana angulata* (Standard Sample); 3. *Magallana hongkongensis* (Standard Sample); 4. *Magallana sikamea* (Standard Sample); 5. Blank Control; The remaining wells contain the tested samples. 6. *M. sikamea*; 7. *M. sikamea*; 8. *M. sikamea*; 9. *M. sikamea*; 10. *M. sikamea*; 11. *M. sikamea*; 12. *M. sikamea*; 13. *M. sikamea*; 14. *M. sikamea*; 15. *M. sikamea*.

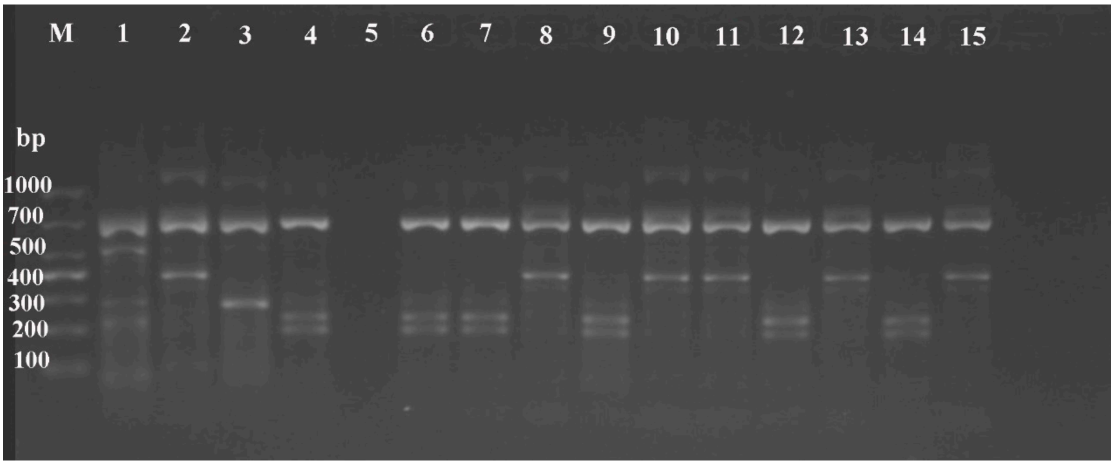

Figure S2. Multiplex species-specific PCR identification results of large oysters. Note: M. 1000 bp DNA Marker; 1. *Magallana sikamea* (Standard Sample); 2. *Magallana hongkongensis* (Standard Sample); 3. *Magallana ariakensis* (Standard Sample); 4. *Magallana angulata* (Standard Sample); 5. Blank Control. The remaining wells contain the tested samples. 6. *M. angulata*; 7. *M. angulata*; 8. *C. hongkongensis*; 9. *M. angulata*; 10. *M. hongkongensis*; 11. *M. hongkongensis*; 12. *M. angulata*; 13. *M. hongkongensis*; 14. *M. angulata*; 15. *M. hongkongensis*.
